# 

**DOI:** 10.1017/S0950268818002297

**Published:** 2018-09-28

**Authors:** Philip Mortimer

The last wild case of smallpox occurred in Somalia in 1977, but due to the escape of a laboratory virus at the Birmingham University Medical School the following year the UK experienced two later cases, one of them fatal. Forty years on, before memories have entirely faded, Mark Pallen has gathered together the evidence surrounding that event. He offers a new explanation for the escape, one from which an important lesson can be drawn. Pallen's ‘Last days of smallpox’ may be read as an interesting tale, which it is, but it also acts as a warning to scientists not to allow personal involvement in any related field, such as emergent pathogens, to cloud their judgement with regard to safety.

The main setting of the book is Birmingham and the year 1978. There had not been autochthonous smallpox in the UK since 1906 or any significant importation of the virus since 1962; yet in 1978, when it was already thought that wild smallpox had been eradicated (which it had), a small inadequately funded English university group was still propagating pox viruses for research purposes. The aim was to be able to characterise any pox virus pathogenic for humans that might emerge in the future following the eradication of natural smallpox; but in hindsight that work should already have been abandoned. The rationale for it was doubtful and the laboratory accommodation inadequate.

Pallen notes that the 1978 escape recapitulated two earlier episodes in UK, one in 1966 from the same laboratory in Birmingham, and the other in 1973 originating from a tiny facility for pox virus research at the London School of Hygiene and Tropical Medicine. In both instances circumstances resembling those of 1978 had given rise to similar misadventures. The earlier, less well known, of these involved an infected laboratory worker who caused an extensive outbreak of minor smallpox (alastrim) in the community: that outbreak is usefully described in Pallen's book. The London smallpox escape in 1973 led to two deaths from major smallpox, however, and considerable disruption in the community. It was competently investigated at the time (see the Cox Report, HMSO, 1974), but without regard to its wider implications. As far as Cox was concerned, at a time when the World Health Organisation was evidently well on the way to eradicating smallpox, a metropolitan laboratory was continuing to propagate its virus without due care and for poorly defined purposes.

Professor Pallen then analyses the inquiry and subsequent court case that followed the later, 1978, release of smallpox virus in Birmingham. The Shooter inquiry concluded that an aerial escape of smallpox virus via ventilation ducting infected a woman who worked in a different university department one floor above, and this explanation was not overturned in court. Shooter and colleagues found much else to criticise, but uppermost in their minds seems to have been their wish to prevent any further research on a dangerous pathogen being undertaken in an inherently unsafe environment within a multi-purpose building, in particular one sited in an urban area.

This was a valuable consequence of the Shooter inquiry even if its main finding of aerial spread of virus has remained open to question. Forty years on a close observer from 1978 implied to Professor Pallen that, just as at the School of Hygiene in 1973, the first Birmingham case in 1978 was probably infected not by a stray air current, but because she had gone into one of the pox laboratories. One or more unauthorised visit may have taken place though no one offered evidence of this at the time and it was impossible to ask Mrs Janet Parker directly. Although she was said to have been vaccinated 11 years earlier, as a photographer having contact with chemicals her skin may have been especially vulnerable. After an unusually prolonged illness she had died of smallpox.

For the general reader, Pallen's book is by no means too heavyweight, and it contains a salutary lesson for professionals. The safety of employees, their families and the wider community is not negotiable. Nonetheless, the current concern about the emergence of new human pathogens and the anxiety that smallpox might itself yet emerge as a bio-terrorist weapon justifies the existence of a few bio-secure facilities worldwide for the study of dangerous pathogens. Beyond that, if the cost of installing appropriate microbiological containment cannot be met or strict working protocols not enforced ongoing research work has to be abandoned and diagnostic inquiries diverted elsewhere.

More generally, it may be recalled that the events of 1978 were contemporaneous with the establishment of a Communicable Disease Surveillance Centre (CDSC) for England, and an equivalent for Scotland. They were both arms-length from Government, allied to national laboratory networks and competent in early outbreak recognition and subsequent case finding. It was no longer acceptable for the response to an outbreak of an unusual pathogen, whether of UK origin or imported, to be left entirely in local hands, however competent those might be. Incubating cases of infection can quickly disperse themselves nationwide and it needs a national facility to deal with that. In subsequent years, CDSC has enhanced the response to ‘new’ pathogens such as Legionnaires’ disease and norovirus, and to imported emergent infections whether rare like Lassa fever or pervasive like HIV. Every country needs one!

